# Alterations of the cytoskeleton in human cells in space proved by life-cell imaging

**DOI:** 10.1038/srep20043

**Published:** 2016-01-28

**Authors:** Thomas J. Corydon, Sascha Kopp, Markus Wehland, Markus Braun, Andreas Schütte, Tobias Mayer, Thomas Hülsing, Hergen Oltmann, Burkhard Schmitz, Ruth Hemmersbach, Daniela Grimm

**Affiliations:** 1 Department of Biomedicine, Aarhus University, 8000 Aarhus C, Denmark; 2 Clinic for Plastic, Aesthetic and Hand Surgery, Otto-von-Guericke University, 39120 Magdeburg, Germany; 3 Deutsches Zentrum für Luft- und Raumfahrt (DLR), Raumfahrtmanagement Bonn-Oberkassel, Bonn, 53227 Germany; 4 Institute for Molecular Physiology and Biotechnology of Plants (IMBIO), Gravitational Biology Group, University of Bonn, Bonn, 53115 Germany; 5 Airbus Defense and Space, Airbus DS GmbH, 28199 Bremen, Germany; 6 FEI Munich GmbH, 82166 Gräfeling, Germany; 7 DLR German Aerospace Center, Department of Gravitational Biology, 51147, Köln, Germany

## Abstract

Microgravity induces changes in the cytoskeleton. This might have an impact on cells and organs of humans in space. Unfortunately, studies of cytoskeletal changes in microgravity reported so far are obligatorily based on the analysis of fixed cells exposed to microgravity during a parabolic flight campaign (PFC). This study focuses on the development of a compact fluorescence microscope (FLUMIAS) for fast live-cell imaging under real microgravity. It demonstrates the application of the instrument for on-board analysis of cytoskeletal changes in FTC-133 cancer cells expressing the Lifeact-GFP marker protein for the visualization of F-actin during the 24^th^ DLR PFC and TEXUS 52 rocket mission. Although vibration is an inevitable part of parabolic flight maneuvers, we successfully for the first time report life-cell cytoskeleton imaging during microgravity, and gene expression analysis after the 31^st^ parabola showing a clear up-regulation of cytoskeletal genes. Notably, during the rocket flight the FLUMIAS microscope reveals significant alterations of the cytoskeleton related to microgravity. Our findings clearly demonstrate the applicability of the FLUMIAS microscope for life-cell imaging during microgravity, rendering it an important technological advance in live-cell imaging when dissecting protein localization.

Although studies on adherently growing human cells exposed to short-term real microgravity during parabolic flight maneuvers prior to fixation and subsequent analysis on Earth have provided some evidence of cytoskeleton alterations^1^,^2^,^3^,^4^,^5^, in-flight live-cell imaging has not been performed. To overcome this obstacle, we have developed a spinning-disc Fluorescence Microscopy Analysis System (FLUMIAS) and investigated cytoskeletal changes during Parabolic Flight Campaigns (PFCs) on-board the Airbus A300 ZERO-G and during the TEXUS 52 sounding rocket mission in stable transfected human follicular thyroid carcinoma cells (FTC-133) expressing the Lifeact-GFP fusion protein for the visualization of F-actin.

Long-term spaceflights have an enormous impact on human health^6^. Several health problems have been reported, such as muscle atrophy, bone loss, cardiovascular problems, among others^6^. The immune system is also altered by the microgravity environment, resulting in immunosuppression in space^6^. A large proportion of the immune cells are compromised and the secretion of cytokines is changed^7^. Changes in the vimentin cytoskeleton were induced in Jurkat cells – a T-lymphoid cell line – by real microgravity (in a Maxus rocket flight)^8^. Another study showed that J-111 monocytes exposed to low gravity conditions exhibited reduced fluorescence intensity of F-actin fibres^9^.

A variety of cellular alterations have been observed after short-term and long-term culture of cells under conditions of simulated and real microgravity^10^,^11^,^12^,^13^,^14^,^15^. Adherently growing human cancer cells and benign cells, which grow normally under static 1 *g*, can be induced to grow three-dimensionally when they are cultured under microgravity conditions. They change their protein content and secretion, as well as displaying differential gene expression^12^. Our understanding of the fundamental role of gravity in cancer cell growth and function is a new paradigm in cell biology^10^,^11^. Notably, changes in the cytoskeleton have been observed in different types of cells such as chondrocytes, lymphocytes, glial cells, breast cancer cells, endothelial cells, and thyroid cancer cells^1^,^3^,^5^,^16^,^17^,^18^,^19^,^20^. These experiments performed in microgravity showed changes in morphology, cytoskeleton and function. Studies on microtubules in altered gravity conditions have shown that they also are gravity-sensitive^18^,^21^,^22^,^23^.

We aim to extend our knowledge in understanding the biology of cancer by using microgravity as a new method, which may be useful to detect interesting proteins, which may become a future target. Hence, to investigate changes in the cytoskeleton of cancer cells during microgravity conditions the compact spinning-disc FLUMIAS device for live-cell imaging under real microgravity was developed and used for on-board analysis in FTC-133 cancer cells expressing the Lifeact-GFP marker protein. It allowed us the visualization of F-actin during a Deutsches Zentrum für Luft- und Raumfahrt (DLR) PFC and the DLR TEXUS 52 sounding rocket mission. The aircraft and also the TEXUS sounding rocket fly parabolic trajectories that produce a period of free fall (microgravity). The term microgravity in general refers to the still existing residual accelerations. For parabolic flights (PF) the free fall phase persists for 22 seconds in which the experiments will experience microgravity in the range of ~10^−2^ *g*^1^,^3^,^4^,^5^,^15^,^24^. For the TEXUS 52 rocket flight the payload is in free fall for a period of up to 390 seconds with a high quality of microgravity (<10^−4^ *g*). Hypergravity and vibrations are inexorable events of PF and spaceflights appearing prior and after the free fall phase which need to be taken into consideration, when evaluating the impact of microgravity^1^.

## Results

### Development of the FLUMIAS microscope

**Figure 1:**
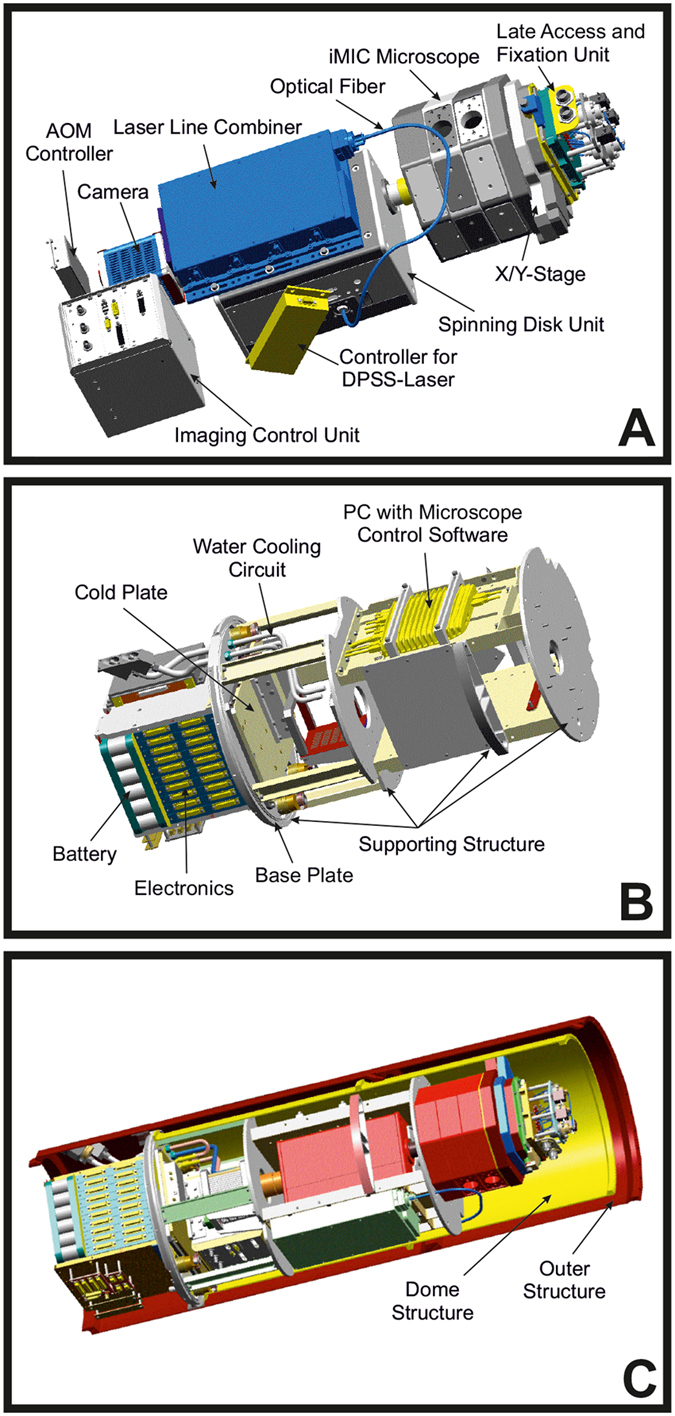
The FLUMIAS experiment module. (**A**) Main components of the inverted confocal laser spinning disc fluorescence microscope with late access and fixation unit. (**B**) Experiment service subsystem for the FLUMIAS TEXUS 52 FM. (**C**) The FLUMIAS experiment module with dome structure integrated into the outer structure.

Two versions of the FLUMIAS microscope module have been built (Fig. 1) and used on the 24^th^ DLR PFC and the DLR TEXUS 52 sounding rocket mission (Supplementary Figure S1A,B). The first one as an engineering model (EM) was used for functional verification and mechanical qualification of FLUMIAS. Furthermore, it is suitable for PFCs and ground support tasks (Supplementary Figure S1C). The second one is the actual TEXUS 52 flight model (FM) (Supplementary Figure S1D). Both versions are identical with respect to the microscope core components and differ only in the way the experiment service subsystem has been implemented. The heart of the FLUMIAS experiment module is the confocal laser spinning disc fluorescence microscope which allows for the parallel scanning of thousands (1200/FOV) of sample points resulting in fast image creation. This set-up was chosen in order to obtain thin slices by eliminating the contribution of out-of-focus light in each image plane, rather than actual physical sectioning. Referred to as optical sectioning, the resulting image planes provide a high level of contrast with improved signal-to-noise ratio, which is equally important for real-time cell monitoring during operation, especially on PFCs, and subsequent data analysis.

The FLUMIAS confocal laser spinning disc fluorescence microscope was developed by FEI Munich GmbH, Germany (Fig. 1). Figure 1A shows the key components of the confocal laser spinning disc fluorescence microscope. The FEI iMIC was a good fit to the space requirements and was used as the main microscope body. Due to the restricted available space, the alignment of the components had to be adjusted and some components had to be re-designed to be more compact so that they could fit into the rocket. To withstand the vibrations and high accelerations during the ascent of the sounding rocket, the number of moveable parts (such as the filter slider and the nosepiece) was reduced to a minimum. The cells were seeded onto an Ibidi-slide (see Methods), which was installed inside the late access and fixation unit (designed and built by Airbus DS, Supplementary Figure S1E). The late access and fixation unit could be separately mounted to the FLUMIAS experiment module. Hence, it was possible to hand over the cells shortly (around two hours) before lift-off. The unit is temperature-controlled and allowed the chemical fixation of the cells under investigation at any time point during the flight. Due to guiding elements, a positional stability of about 10 μm in the x- and y-directions for the demounting-/mounting-process was achieved. The late access and fixation unit was mounted on top of the X/Y-stage, allowing movement in the x- and y-direction (around 24 mm travel range in both axes). This travel range allowed the observation of three out of the six channels of the Ibidi slide during the flight. The X/Y-stage is directly attached to the octagonal base of the microscope body, the iMIC. The iMIC contained the microscope objective (Carl Zeiss, Plan-Apochromat 40x/0.95 Corr), which was mounted on a voice coil focus drive for movements in z-direction. The spinning disc unit (called Andromeda) is connected to the iMIC and to the camera and besides the spinning disc contained several optical elements necessary for beam shaping of the incoming laser light. The laser light originated from the laser line combiner (Omicron-Laserage Laserprodukte) and was coupled into the spinning disc unit via an optical multimode fiber. In the current configuration four different excitation wavelengths could be chosen out of three diode lasers (405 nm/120 mW, 488 nm/200 mW and 642 nm/140 mW) and one diode-pumped solid state (DPSS) laser (561 nm/150 mW). While the diode lasers could be easily switched on and off very fast, the DPSS laser could only be indirectly switched using an acousto-optical modulator (AOM), which needed an individual controller. The initialization and interaction of all components is managed by the imaging control unit (ICU), which also supplied power to the iMIC and the X/Y-stage.

As a supporting structure and for power supply, cooling and control of the microscope, an experiment service subsystem (Airbus DS) was built for the FLUMIAS TEXUS 52 FM, which is depicted in Fig. 1B. The experiment service subsystem can be roughly separated in two parts. The first part below the base plate contained the battery and the electronics (DC/DC converter, experiment timer, etc.). This part experienced vacuum during the flight. The second part above the base plate was shielded with a dome structure (Fig. 1C) against vacuum and besides the supporting structure contained a water-cooling circuit and a PC with microscope control software (an adapted release of the software package L.A. by FEI Munich GmbH) and storage for the high-resolution images. Before flight the FLUMIAS module was integrated into the dome structure (Fig. 1C) and stacked with other experiment modules to form the TEXUS 52 payload (Supplementary Figure S1F). To regulate the module temperature inside the dome structure, the camera and the cold plate were connected to the water-cooling circuit. The ICU, being the main heat source, was directly mounted onto the cold plate. During lift-off the cooling circuit was automatically disconnected.

**Figure 2:**
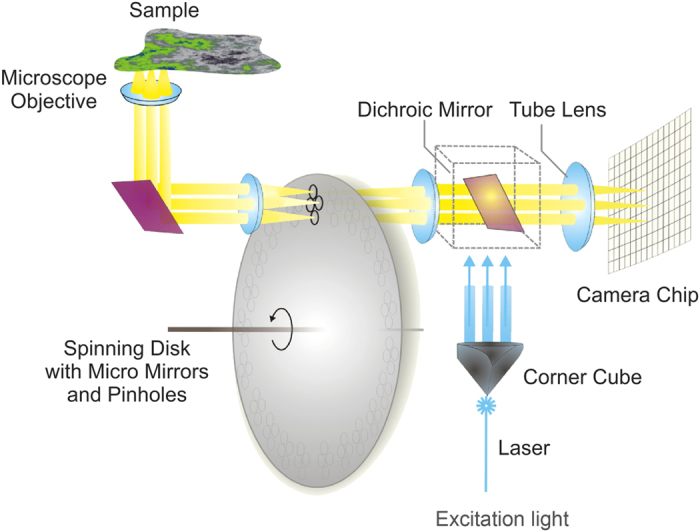
Sketch of the working principle of a confocal laser spinning disc fluorescence microscope. A laser beam is coupled through a multimode fiber into the spinning disk system. The beam is focused through a hole in the corner cube and sent collimated towards the disk with pinholes. Micro mirrors around the pinholes reflect a beam bundle back into the corner cube. Each sub-beam is retro-reflected and focused through the pinholes. The light passes the objective and is focused on the sample plane, where it generates fluorescence in the sample. The fluorescence emission is focused though the pinholes and transmits the dichroic mirror towards the camera chip.

A sketch depicting the working principle of a confocal laser spinning disc fluorescence microscope is shown in Fig. 2. The laser light (excitation light) enters the spinning disc unit through a small hole in the corner cube and is reflected by a dichroic mirror onto the spinning disc. In the first run only a small amount of light passes the pinholes, but the majority of light is reflected by the concave micro-mirrors back to the dichroic mirror. After taking the path from the dichroic mirror to the corner cube and the dichroic mirror, the light again reaches the spinning disc but this time most of the light passes the pinholes due to the effect of the micro-mirrors and is focused onto the sample. Based on the confocality only fluorescent light originating from the focal plane in the sample can pass the pinholes of the spinning disc and reach the camera chip, after transmission by the dichroic mirror (the emission wavelength is distinctly larger than the excitation wavelength).

The confocal laser spinning disc fluorescence microscope offers a high axial resolution (around 1.5 μm) compared to normal light microscopes by efficiently suppressing the fluorescent light, which does not originate from the focal plane. The central part of the confocal fluorescence microscope is the spinning disc, which contains many pinholes (approximately 1200) and around each pinhole micro mirrors are etched into the substrate (see Fig. 2). The pinholes have a fixed diameter of a few tenths of micrometers and are arranged in a spiral pattern. Due to this special arrangement all pixels of the camera chip are exposed to fluorescence light and accordingly a complete new frame is created whenever the disc is rotated through about 30 degrees (12 frames per 360° rotation). As the spinning disc is rotating with a speed of 5000 rpm, it is theoretically possible to record 1000 frames per second. However, for standard biological samples the exposure time is typically above 50 ms, hence the resulting image is averaged over several frames.

### Live-cell imaging of the cytoskeleton in FTC-133 cells

**Figure 3:**
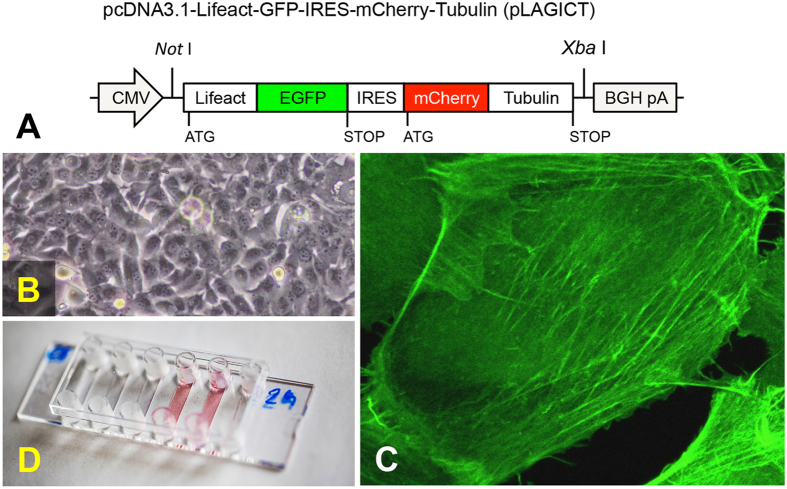
Generation of FTC-133 cells expressing Lifeact-GFP for FLUMIAS-based life-cell imaging. (**A**) Schematic presentation of the versatile pLAGICT plasmid containing the Lifeact-eGFP-IRES-mCherry-Tubulin (LAGICT) expression cassette for visualization of F-actin and α-tubulin. A CMV promoter drives the expression of the LAGICT cassette and efficient transcriptional termination is provided by the bovine growth hormone (BGH) polyadenylation site of the pcDNA3.1(+) vector. For selection purposes the vector contains the neomycin gene driven by a SV40 promoter. (**B**) Bright field image of FTC-133 cells. (**C**) Example of Lifeact-GFP-visualization of F-actin in FTC-133 cells stable transfected with pLAGICT. (**D**) The *μ*-slide VI 0.4 ibiTreat slide assembled with medium containing flexible tubes prior to insertion into the late access and fixation unit.

To extend our knowledge about changes in the cytoskeleton of poorly differentiated follicular thyroid cancer cells during microgravity conditions, live-cell imaging of the cytoskeleton was performed in FTC-133 cells. Importantly, these cells had been investigated in space twice previously^4^,^14^,^25^ and they are very robust, when cultured under microgravity especially during launch^4^,^14^,^25^. Interestingly, the cells have developed a less-aggressive phenotype during one of the space missions^4^, so exploring these changes induced by microgravity may lead to the identification of new targets for cancer therapy. Furthermore, they were investigated on PFCs^4^ and have been thoroughly analyzed in simulated microgravity^12^,^25^,^26^ making FTC-133 cells to one of the best-characterized cell types, cultured under altered gravity conditions. In addition, they can be easily transfected. The cells were stably transfected with a pcDNA3.1(+)-based vector, entitled pLAGICT, expressing Lifeact-GFP for the visualization of F-actin (Fig. 3A). The vector also encodes mCherry-Tubulin fusion proteins enabling parallel analysis of both actin and tubulin counterparts of the cytoskeleton. However, due to the experimental setup only live-cell imaging of Lifeact-GFP was performed during microgravity. Following transfection with pLAGICT and G418 selection, resistant FTC-133 clones (Fig. 3B) were isolated and inspected by fluorescent microscopy. A clone (designated Lifeact-GFP) with bright Lifeact-GFP labeling of F-actin was chosen and further expanded (Fig. 3C).

### The FLUMIAS microscope is functional in microgravity during parabolic flights maneuvers

**Figure 4:**
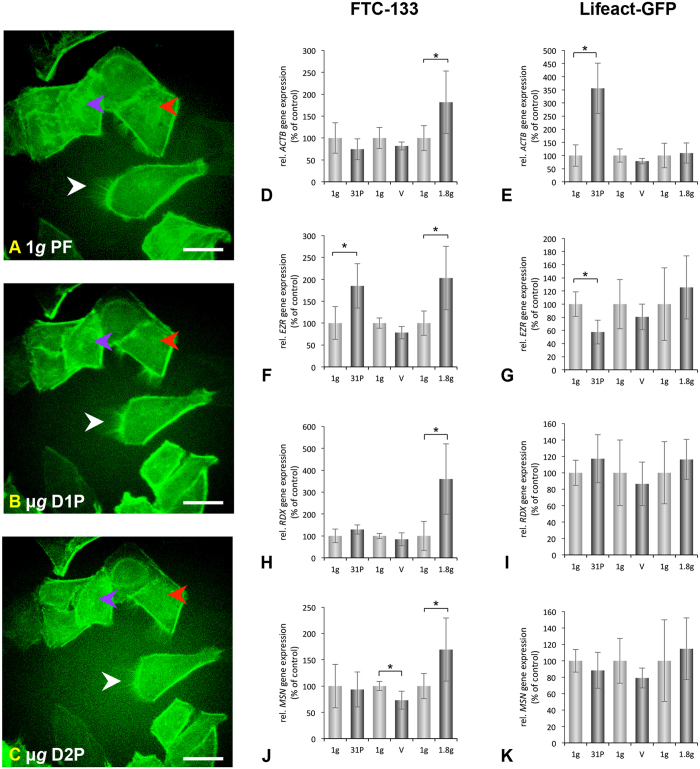
Inflight analyses of cytoskeletal changes during the PFC. (**A**–**C**) Imaging of F-actin visualized by Lifeact-GFP using the FLUMIAS EM. (**D**–**K**) Gene expression analysis assessed by RT qPCR to investigate changes in the cytoskeleton. FTC-133 cells expressing Lifeact-GFP were cultivated in *μ*-slides for life-cell imaging. The position of cells displaying Lifeact-GFP fluorescence signals was selected and life-cell imaging of these selected cells was performed both pre-flight (**A**) as well as during the microgravity phase of the 1 parabola (D1P) (**B**) and the second parabola (D2P) (**C**). Scale bars = 20 μm. Purple arrowheads indicate cytoskeleton alterations in the form of “holes” in the cytoplasm. Red arrowheads indicate disturbance of F-actin bundles. White arrowheads indicate disappearance of microvilli or filopodia-, and lamellipodia-like structures. For gene expression analysis non-transfected FTC-133 cells and FTC-133 cells expressing Lifeact-GFP were cultivated in T175 cell culture flasks. To rule out the effect of vibration and hyper-*g*, which are obligatory events occurring both before and after each parabola, cell cultures were independently subjected to either vibration experiments using a Vibraplex device or hyper-*g* experiments using a short-arm human centrifuge with corresponding ground controls (1 *g*) as described in Methods. (**D**,**E**) Gene expression of *ACTB* in FTC-133 and cells expressing Lifeact-GFP, respectively after 31 parabolas (31P), vibration (V), and hyper-*g* (1.8 *g*) with corresponding ground controls (1 *g*). (**F**,**G**) Gene expression of *EZR* in FTC-133 and cells expressing Lifeact-GFP, respectively after 31 parabolas (31P), vibration (V), and hyper-*g* (1.8 *g*) with corresponding ground controls (1 *g*). (**H**,**I**) Gene expression of *RDX* in FTC-133 and Lifeact-GFP, respectively after 31 parabolas (31P), vibration (V), and hyper-*g* (1.8 *g*) with corresponding ground controls (1 *g*). (**J**,**K**) Gene expression of *MSN* in FTC-133 and Lifeact-GFP, respectively after 31 parabolas (31P), vibration (V), and hyper-*g* (1.8 *g*) with corresponding ground controls (1 *g*). All results are shown as mean ± standard deviation (SD) of n = 8 (PFC), n = 12 (hyper-*g*) and n = 5 (vibration) independent samples, with significance indicated by *P < 0.05 vs. 1 *g*.

During a parabolic flight, 31 consecutive parabolas were performed. One parabola contains three phases (pull up, free fall, and pull out). A parabola starts from the horizontal flight level followed by a 45° ascent for 20 seconds in which experiments and the passengers experience hypergravity in the range of 1.5–1.8 *g*. Following a reduction in the thrust, the aircraft follows the trajectory of a parabola initiating the 22-second-long free fall (or microgravity) phase. Finally, the engines are powered up again, and a second phase of 1.8 *g* for 20 seconds terminates the parabola. Due to turbulence acting on the aircraft as well as the manual operation of the aircraft, the microgravity is in the range of ~10^−2^ *g*^1^,^3^,^4^,^5^,^15^,^24^. Prior to each flight an Ibidi-slide (Fig. 3D) with FTC-133 cells expressing Lifeact-GFP seeded into it was installed inside the late access and fixation unit (Supplementary Figure S1E) and brought to the aircraft. The live-cell imaging results obtained on-board the Airbus A300 ZERO-G indicated that disturbances of actin bundles and “holes” within the cytoplasm, appeared immediately during the μ*g* phase of parabola 1 in cells expressing Lifeact-GFP when the cells were cultivated adherently on slides (compare Fig. 4A,B). This process seemed to develop during the following parabola (Fig. 4C). Conversely, no “holes” were observed in the cytoplasm of the cells expressing Lifeact-GFP before parabola 1 (Fig. 4A). Moreover, the analysis indicated the disappearance of microvilli or filopodia-, and lamellipodia-like structures during the parabolic flight (Fig. 4A–C). Taking into account that the “holes” are considered to indicate points of cell cytoplasm discontinuity, the results clearly indicate that the cytoskeleton of low-differentiated follicular thyroid cancer cells is not resistant to a fast and short removal of the influence of gravity for 20 seconds, and importantly, that the cytoskeletal changes occur rapidly after entrance into the μ*g*-phase of parabola 1. The finding that the cytoskeletal alterations occur after only 1 parabola is supported by our previous findings^3^,^5^. However, by using the FLUMIAS-based live-imaging approach during PFCs, we are now able to dissect the point of action to be in the μ*g*-phase. No apoptotic or dead cells as judged by visual inspection of the cells at the end of each of the individual experiments were identified in the 1*g*-controls on the ground or in the cells exposed to the parabolic flight profile.

To test whether the observed changes in the cytoskeleton during the parabolic flight maneuvers may reflect transcriptional alterations, *ACTB*, *EZR*, *RDX* and *MSN* gene expressions after 31 parabolas were measured. *ACTB* expression was not altered during the parabolic flight, but a 2-fold increase was observed during hyper-*g* in non-transfected cells (Fig. 4D). The expression of *ACTB* was found to be increased 3.5-fold in cells expressing Lifeact-GFP exposed to parabolic flight maneuvers compared to 1 *g* control cells (Fig. 4E). No changes in the expression of *ACTB* were detected during vibration and hyper-*g* in cells expressing Lifeact-GFP (Fig. 4E). This observation might either be related to the integration site(s) of the expression pLAGICT cassette or to the fact that the cells expressing Lifeact-GFP were exposed to a selection procedure.

The proteins ezrin, radixin and moesin (ERM) are known to crosslink the plasma membrane and the actin cytoskeleton^27^. By this procedure, they provide both structural links to strengthen the cell cortex and control signal transduction pathways. Hence, the ERM proteins are involved in membrane dynamics, adhesion, cell survival, cell motility and morphogenesis^27^. Despite the overall similarity in function and structure, individual functions of the three proteins appear to be specialized^27^. Notably, there is evidence that ERM proteins are involved in the regulation of tumor progression and metastasis. Ezrin functions as a protein-tyrosine kinase substrate in microvilli^28^ and is frequently overexpressed in metastatic tumor cells^29^. The ezrin gene was up-regulated in established anaplastic thyroid carcinoma cells^30^. Radixin functions as a membrane-cytoskeletal crosslinker in actin-rich cell surface structures^27^ and it is reported that the expression level of radixin is found to be significantly unregulated in colon tumor tissues^31^. Moesin is phosphorylated at the site of entry of mitosis and is involved in several important steps throughout cell division^32^. Inactivation of moesin disrupts spindle organization. It acts as a potential marker in breast and pancreatic cancer, and the expression level of moesin is linked to tumor development of oral squamous cell carcinoma^33^.

In general little is known about ERM proteins and thyroid cancer. We recently had shown that the secretion of ezrin by FTC-133 cells is increased after 10 days in space^4^. Here we detected an increase of *EZR* mRNA after only 31 parabolas, indicating an early signaling process (Fig. 4F). This *EZR* mRNA increase may also be induced by hyper-*g*. This finding could be confirmed with the ML-1 cell line, in which *EZR*, *RDX* and *MSN* mRNAs were also increased by hyper-*g*^34^. In endothelial cells *EZR* mRNA was up-regulated after 31 flown parabolas, a finding corresponding well to the results found for FTC-133 cells.

Overall, no significant changes in the expression of the *EZR*, *RDX* and *MSN* genes in cells expressing Lifeact-GFP compared to the ground control cells were detected (Fig. 4G,I,K), except that during the PFC *EZR* expression was reduced by approximately 40% (Fig. 4G). In the FTC-133 cells we found that *EZR* expression was increased approximately 2-fold during both PFC and hyper-*g* compared to 1 *g* (Fig. 4F). Similarly, the *RDX* expression was increased 3.5-fold during hyper-*g* (Fig. 4H). *MSN* expression was also increased during hyper-*g*, whereas *MSN* expression appeared to be reduced by 20% during vibration compared to 1 *g* (Fig. 4J).

**Figure 5:**
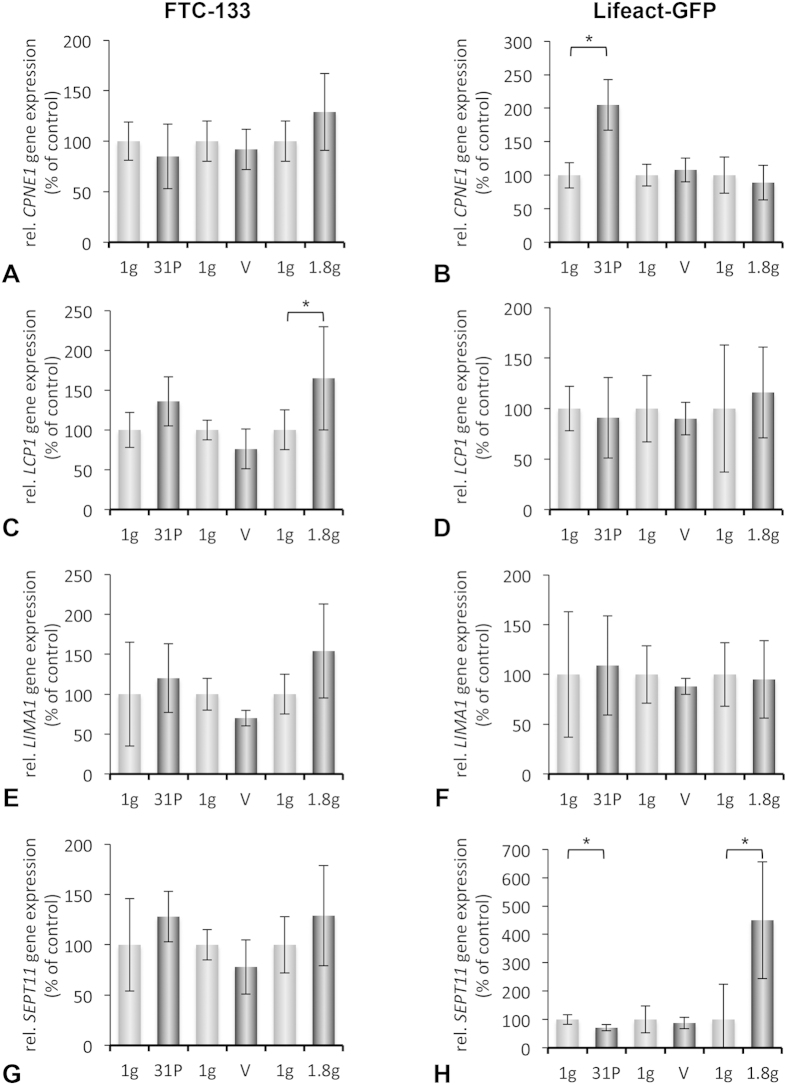
Gene expression analysis evaluated by RT qPCR to investigate the changes of proteins regulating cellular signaling processes. Non-transfected FTC-133 cells and FTC-133 cells expressing Lifeact-GFP were cultivated in T175 cell culture flasks and brought to the aircraft approximately one hour prior take off. To rule out the effect of vibration and hyper-*g*, vibration experiments or hyper-*g* experiments with corresponding ground controls (1 *g*) were performed as described in Methods. (**A**,**B**) Gene expression of *CPNE1* in FTC-133 and cells expressing Lifeact-GFP, respectively after 31 parabolas (31P), vibration (V), and hyper-*g* (1.8 *g*) with corresponding ground controls (1 *g*). (**C**,**D**) Gene expression of *LCP1* in FTC-133 and cells expressing Lifeact-GFP, respectively after 31 parabolas (31P), vibration (V), and hyper-*g* (1.8 *g*) with corresponding ground controls (1 *g*). (**E**,**F**) Gene expression of *LIMA1* in FTC-133 and Lifeact-GFP, respectively after 31 parabolas (31P), vibration (V), and hyper-*g* (1.8 *g*) with corresponding ground controls (1 *g*). (**G**,**H**) Gene expression of *SEPT11* in FTC-133 and Lifeact-GFP, respectively after 31 parabolas (31P), vibration (V), and hyper-*g* (1.8 *g*) with corresponding ground controls (1 *g*). All results are shown as mean ± standard deviation (SD) n = 8 (PFC), n = 12 (hyper-*g*) and n = 5 (vibration) independent samples, with significance indicated by *P < 0.05 vs. 1 *g*.

Moreover, we focused on four proteins regulating cellular signaling processes: Copine 1, plastin 2, sept11 (septin-11) and LIMA. Copine 1 can bind several intracellular proteins with diverse biological functions^35^, its role in regulating biological processes remains unclear. In mammalian cell lines, copines 1, 2, 3, 6 and 7 can move to the plasma membrane following increases in intracellular Ca^2+^ triggered by ionomycin treatment of cells in medium containing 1.8 mm calcium. Copine 1 is a calcium-dependent membrane-binding protein regulating signaling at the cell membranes^36^. The FTC-133 cell line is *CPNE1*-positive and we could demonstrate for the first time in Fig. 5A,B that real microgravity increases *CPNE1* mRNA after the 31^st^ parabola in Lifeact-GFP transfected cells, whereas hyper-*g* and vibration had no effect on this gene.

We recently had detected lymphocyte cytosolic protein 1 (LCP-1 or plastin 2) by proteome analysis in FTC-133 poorly differentiated follicular thyroid cancer cells^37^ and demonstrated that LCP-1 protein was down-regulated in FTC-133 cells cultured in simulated microgravity on the Random Positioning Machine (RPM) for 3 days^38^. LCP-1 is an actin-binding protein and had been earlier identified as an ovarian cancer tumor biomarker^39^. During a PFC we detected a slight increase in *LCP1* gene expression after 31 parabolas and also an increase in hyper-*g* samples. Vibration had no effect on the expression of *LCP1* (Fig. 5C).

Recently, we had investigated ML-1 thyroid cancer cells on a PFC^5^. We found that *LIMA1* mRNAs were slightly, not significantly up-regulated under microgravity, but up-regulated in hyper-*g* after 31 parabolas and unchanged by vibrations. Interestingly, no significant changes were observed when investigating the FTC-133 cell line, but an increase in *LIMA1* mRNA in hyper-*g* samples was detectable (Fig. 5E,F). The LIMA1 protein is a cytoskeletal-associated protein involved in the regulation of actin dynamics and cell motility. It is known to suppress actin depolymerization and to stabilize F-actin fibers^40^. This results in the establishment of the adhesion belt^40^. The up-regulation of *LIMA1* mRNA under hyper-*g* indicates a mechanism of the thyroid cancer cells to stabilize the actin cytoskeleton.

SEPT11 (septin-11) is a GTP-binding protein organized in filaments and was detected in FTC-133 thyroid cancer cells^37^. It is involved in cytokinesis and in microtubule/actin cytoskeleton organization^41^. In transfected cells real microgravity significantly reduced the gene expression of *SEPT11* after 31 parabolas, whereas conditions of hyper-*g* significantly elevated its expression and vibration did not change *SEPT11* (Fig. 5G,H).

### Microgravity during a sounding rocket flight changes the cytoskeleton

**Figure 6:**
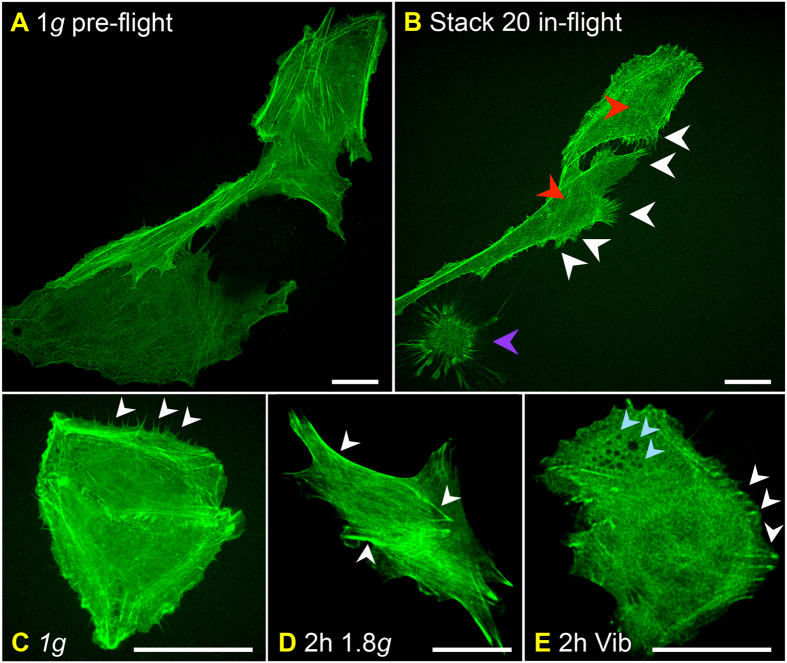
Inflight analyses of F-actin cytoskeleton changes during the TEXUS 52 mission using the FLUMIAS FM. Live-cell imaging of F-actin visualized by Lifeact-GFP in FTC-133 cells experiencing (**A**,**B**) microgravity during the TEXUS 52 rocket flight, (**C**) normal gravity (1 *g*), (**D**) hypergravity (1.8 *g*), or (**E**) vibration. Two hours before lift-off a *μ*-slides containing Lifeact-GFP expressing FTC-133 cells was mounted in the late access and fixation unit and transferred to the rocked as described in Methods. The position of three cells displaying Lifeact-GFP fluorescence signals was selected and live-cell imaging of these cells was performed both before lift-off (**A**) and during the 6-min-long microgravity phase of the flight (**B**). As controls live-cell imaging was performed on cells experiencing 1 *g*, two hours of hyper-*g* and two hours of vibration. (**A**) 1 *g* pre-flight. (**B**) Stack 20 in-flight of the same cells shown in (**A**). (**C**) 1 *g* control. (**D**) 2 hours of hyper-*g* (1.8 *g*). (**E**) 2 hours of vibration (Vib). Scale bars = 20 μm. Red arrowheads indicate disturbance of F-actin bundles. In (**B**) white arrowheads indicate appearance of filopodia-, and lamellipodia-like structures. In (**C**) white arrowheads indicate microvilli. In (**D**) and (**E**) white arrowheads indicate stress fibers. Purple arrowhead denotes cellular detachment. Light blue arrowheads indicate cytoskeleton alterations in forms of stress granules in the cytoplasm.

After examining the functionality of the FLUMIAS EM during a PFC, we next investigated the effect of microgravity during a sounding rocket flight. An advantage of such an experiment compared to a PFC is the considerably longer duration of microgravity (approximately 6 minutes) as well as only one period of hypergravity and vibration preceding the microgravity phase of the rocket flight parabola. This experiment was performed on-board the TEXUS 52 rocket using the FLUMIAS FM. A video of the sounding rocket flight mission is shown in Supplementary Video S2. Even though this version of the microscope is equipped with multiple lasers enabling parallel imaging of Lifeact-GFP and mCherry-Tubulin, we chose to only use the 488 nm diode laser for detection of GFP-Lifeact in order to obtain as much information as possible regarding the actin counterpart of the cytoskeleton immediately after the beginning of microgravity and during the 6 minutes of the microgravity phase. Approximately 60 seconds after lift-off of TEXUS-52, live-images from the FLUMAIS FM were received by telemetry allowing a final adjustment of the x, y and z coordinates selected before launch. Live-cell imaging was then performed in five separate rounds of approximately 25 seconds duration resulting in data collection corresponding to four Z-stacks of 28 layers separated by 300 nm. As presented in Fig. 6, a palette of different actin structures was observed. The pre-flight 1*g*-image revealed the actin cytoskeleton of three cells as visualized by Lifeact-GFP expression (Fig. 6A). As expected, well-structured filament-bundles were observed in the cells and stress fibers as well as filopodia- and lamellipodia-like structures were barely visible. Notably, following entrance into microgravity the actin-based cytoskeleton rapidly underwent dramatic changes (Fig. 6B). These changes included the disturbance of F-actin bundles, the appearance of filopodia- and lamellipodia-like structures and cellular detachment. To obtain information of the dynamics of these changes, a live-cell imaging video was assembled from corresponding sections. The video was created from the z-stacks over the total observation time of approximately 125 seconds (see Supplementary Video S1). In support of the data presented in Fig. 6A,B, the changes, especially the formation of filopodia- and lamellipodia-like structures, occurred rapidly after entrance into the microgravity phase. The appearance of filopodia- and lamellipodia-like structures was most likely a combined result of microgravity and vibration. The structures observed in the 1*g-*ground control cells (Fig. 6C) most likely represent microvilli, which then disappear during hypergravity (Fig. 6D). In addition, stress fibers can then be observed in the hypergravity sample, while microvilli and filopodia- as well as lamellipodia-like structures disappeared (Fig. 6D). In response to 2h-vibrations stress granules appeared as presented in Fig. 6E. Stress fibers could also be observed in response to vibrations (Fig. 6E).

## Discussion

The aim of this study was to investigate the influence of real microgravity on the cytoskeleton on human thyroid cancer cells. For this purpose, we developed a compact fluorescence microscope (FLUMIAS) for fast live-cell imaging under real microgravity. To our knowledge, this is the first report of such a live-cell imaging analysis in space on a TEXUS rocket flight or onboard a parabolic flight aircraft. We used the poorly differentiated thyroid cancer cell line FTC-133 as our cell model system, which was already studied two-times in space (Sino-German Shenzhou-8/SIMBOX space mission in 2011 and NanoRacks-Cellbox-1 ISS experiment in 2014). For this purpose, we cultivated FTC-133 cells for the TEXUS 52 experiment in Kiruna, Swedish Space Center, ESRANGE, Sweden. In addition, we also cultivated the cells under altered gravity conditions on a short-arm human centrifuge and a Vibraplex device (both constructed by DLR, Cologne, Germany) and exposed them to short-term real microgravity on parabolic flights.

A long-term spaceflight has enormous impact on the health of humans in space. Several health problems can occur such as heart problems, bone loss, muscle atrophy, disturbances of the immune system, and more^6^,^42^. Since many years we know that microgravity induces a variety of changes in cells and plants cultured under real and simulated microgravity conditions^2^. Researchers demonstrated changes in growth behavior, differentiation, proliferation, cell adhesion, migration as well as increases in programmed cell death and changes in the cytoskeleton as well as elevated amounts of extracellular matrix proteins in many different cell types cultured under microgravity conditions^11^,^18^,^19^,^43^,^44^,^45^,^46^,^47^,^48^,^49^,^50^,^51^.

Simulated microgravity (or functional weightlessness) is based on the assumption that sensing no weight and neutralization of sedimentation would have effects comparable to those of weightlessness^52^ (0 *g*), a condition which is never completely reached. Therefore, the term “microgravity” is used to indicate the very weak residual acceleration forces. On ground, different facilities have been developed aiming to achieve functional weightlessness (simulated microgravity), such as RPMs or fast-rotating clinostats. Even though RPMs and clinostats have been shown to imitate microgravity responses consistently for several, but not all, experimental conditions, they generally seem to underestimate the effects observed in real microgravity during spaceflights^53^. Therefore, the development of a fluorescence microscope for live-cell imaging studies on different types of cells suitable to perform analyses during parabolic flight missions, on rocket flights and on the International Space Station in space is a necessary step to prove the earlier findings on living cells.

Until now live-cell imaging was not possible in space. This is the first study investigating the cytoskeleton in living thyroid cancer cells. In the past and also currently, researchers had to work with samples treated with paraformaldehyde (PFA) or other fixatives to perform fluorescence staining of the cytoskeleton in different cell types, when they conducted their experiments under conditions of real microgravity. The F-actin network can be visualized by rhodamine-phalloidin staining. Potential pitfalls of PFA fixations and immunolabeling artifacts including severe damage of the cellular cytoskeleton and a shrinking cell volume do often receive little attention arguing that immunostaining experiments in dead, permeabilized cells should be accompanied with live-cell imaging when dissecting protein localization^54^. Taking the data from the PFC and the TEXUS mission together, we found that changes in the cytoskeleton occur rapidly after entrance into microgravity, which confirms the results obtained on fixed samples. In both cases we detected a disturbance of F-actin bundles, which was not observed during 1.8 *g* or vibration. Another important finding observed during microgravity was the formation of filopodia- and lamellipodia-like structures, which were not observed during hypergravity. Contrary, formation of stress fibers occurred in the 1.8 *g* experiments, while microvilli, which were observed in 1 *g-* samples, and filopodia- as well as lamellipodia-like structures disappeared. The cytoskeletal alterations in form of ”holes” in the cytoplasm observed during the parabolic flight maneuvers, presumably representing stress granules, were not evident during the TEXUS flight. However, as stress granules are seen in the cells exposed only to vibration, these changes are most likely linked to the vibration events occurring during each parabola. Notably, stress fibers are present in the samples, experiencing vibration.

The disappearance of filopodia-, and lamellipodia-like structures, as observed in Fig. 4A–C might be explained by the fact that they disappear during the two 1.8 *g* phases of each parabola as they are not observed during the 1.8 *g* experiment. The expression data show that *ACTB* is significantly up-regulated in cells expressing Lifeact-GFP during PFC. This up-regulation is most likely a consequence of μ*g*, since expression of *ACTB* is not altered during vibration and hyper-*g*. Conversely, *ACTB* expression is not altered in FTC-133 during the PFC. However, a 2-fold up-regulation is observed during hyper-*g*.

Viable cells *in vitro* use a tension-dependent form of architecture (tensegrity) for the organization and stabilization of the cytoskeleton^55^. The tensegrity model describes how cells might control their shape and mechanics through use of an architectural mechanism. Even though a growing body of evidence suggests that this model accounts for a number of features of living cells including control of shape and physical connections between the chromosomes^55^,^56^, it remains controversial and has a number of limitations^57^. All cytoskeletal filament systems are involved in this special stress (altered gravity) response^55^. Cytoskeletal disruption is an important factor known to increase programmed cell death^58^ in a variety of cells, but not in all cells. Human chondrocytes cultured on the RPM showed cytoskeletal alterations, but no increase in apoptosis^16^,^59^. Programmed cell death was found in thyroid cancer cells^45^ and in adherent EC cultivated on the RPM as well as in Jurkat cells and lymphocytes cultured in space^18^,^43^. This is in agreement with our finding of one dying FTC-133 cell during the rocket flight (Fig. 6B). These data support the hypothesis that the cytoskeletal and ERM proteins seem to be the first proteins influenced by microgravity because their networks of interaction are disturbed in a few seconds when the gravity vector is annulled. Therefore, they cannot longer function in a proper manner. The result is a rapid influence on gene regulation, initiating a cascade of protein changes. Thus, exact knowledge of the proteome in different cell types, together with the gene array technique, is useful to clarify which signaling pathways are involved in cancer or other diseases occurring in space^4^,^25^.

The ERM protein group, consisting of the three closely related proteins ezrin, radixin and moesin, is known to crosslink actin with the cellular plasma membranes. We had detected an accumulation of F-actin at the outer membranes of different cell types so that we focused on changes in these proteins in space and after the PFC. The expression of ERM genes, with the exception of *EZR*, was not altered in cells expressing Lifeact-GFP during the PFC, vibration or hyper-*g*. Increased expression of *EZR* in FTC-133 during the PFC may be caused by hyper-*g* as a similar increase of *EZR* is observed under 1.8 *g* conditions. The increase of *ACTB* in FTC-133 during hyper-*g* was also reflected in an increased expression of *RDX* and *MSN*.

Our observations demonstrate that the cells sense gravitational unloading rapidly after the onset of microgravity, which consequently leads to immediate formation of filopodia-, and lamellipodia-like structures. Since these actin-dependent filaments are involved in cellular processes like adhesion and migration, it makes sense for the cells to make use of such molecular architecture elements^60^. Our experiments also provide data indicating alteration in expression of ERM genes. These proteins strongly localize to microvilli and it has recently been shown that over-activation of ERM proteins leads to tightening of the cortex-membrane linkage as reported by Fritzsche *et al.*^61^. In response to over-expression of ezrin and F-actin, an orchestrated sequence of different actin structures may thus be envisioned for the cells until they experience severe issues characteristic for stress granules^62^.

In conclusion, the FLUMIAS microscope has elegantly proven its applicability for live-cell imaging during both PFC and TEXUS 52 flights and by using this platform, in combination with gene expression analysis, we have documented significant alterations of the cytoskeleton, occurring rapidly after entrance to microgravity. We believe that the concept of using the FLUMIAS live-cell imaging system in microgravity might provide important knowledge in our understanding of the fundamental role of gravity in cell biology including cancer cell growth and function. We also believe that the FLUMIAS microscope is an important technological advance in live-cell imaging when dissecting protein localization.

## Methods

### The FLUMIAS spinning disc microscope

The FLUMIAS confocal laser spinning disc fluorescence microscope was developed by the company FEI Munich GmbH, Germany (Fig. 1, see text for details). A sketch depicting the working principle of a confocal laser spinning disc fluorescence microscope is shown in Fig. 2. The biological samples are seeded to an Ibidi-slide (ibidi GmbH, *μ*-slide VI 0.4 ibiTreat slide) (Fig. 3D), which is installed inside the late access and fixation unit (designed and built by Airbus DS) (Supplementary Figure S1E). The FLUMIAS microscope is equipped with a 40x objective (Carl Zeiss Microscopy GmbH, Plan-Apochromat 40x/0.95 Corr), a spinning disc unit (called Andromeda) and a camera (Hamamatsu Photonics Deutschland GmbH, Orca flash 4.0). The laser light originates from the laser line combiner (Omicron-Laserage Laser products GmbH, LightHUB-4). As a supporting structure and for power supply, cooling and control of the microscope Airbus DS built an experiment service subsystem for the FLUMIAS TEXUS 52 FM.

#### Parabolic flight

The parabolic flight experiments were carried out on-board the Airbus A300 ZERO-G, operated by the Bordeaux-Merignac-based company Novespace (France) (Supplementary Figure S1A). The parabolic maneuvers each consisted of 31 consecutive parabolas during the flight^1^. The procedure was described earlier in detail^1^,^3^,^24^.

#### Cell culture procedure for the PFC

The cell culture procedure for the 24^th^ and 25^th^ DLR parabolic flight campaigns was published earlier^1^,^4^. The human follicular thyroid carcinoma cell line FTC-133 was purchased from the Health Protection Agency Culture Collections (HPACC). The cells were cultivated in RPMI 1640 (Life Technologies) medium supplemented with 10% FCS (Biochrom AG) and 1% penicillin/streptomycin (Life Technologies) and cultured under standard cell culture conditions at 37 °C and 5% CO_2_. In brief, the cells were cultivated in 16 T175 cell culture flasks (175 cm^2^; Sarstedt). During this time, the cells were covered by 15 ml (T175 flasks) of complete medium. Half of the flasks (n = 8) with appropriate medium were used as 1 *g* ground control cells, cultured, and fixed in the laboratory, and the other half were, in parallel, taken to the aircraft for the parabolic flight (n = 8). Syringes containing the appropriate fixative (RNA*later*) were connected to both types of flasks for the parabolic flight via a flexible tube and 3-way valve^1^. One hour before each flight, the cell culture flasks and slides were transported to the aircraft and placed into the 37 °C preheated incubator on an experimental rack. After the flight the samples were immediately brought to the cell culture laboratory at Novespace, Bordeaux-Merignac, France.

#### TEXUS 52 sounding rocket flight mission

The rocket flight experiment was performed on-board a sounding rocket as part of the DLR TEXUS (Technologische EXperimente Unter Schwerlosigkeit) 52 mission launched from Esrange, Swedish Space Center (SSC), in Kiruna in Northern Sweden (see Supplementary Figure S1B and Supplementary Video S2). The rocket type was a Brazilian two-stage solid propellant VSB 30 rocket (Supplementary Figure S1F). The duration of the flight was approximately 15 minutes and the parabolic flight brought the sounding rocket to an altitude of 261 km. Following launch, the rocket enters microgravity after 75 seconds of flight and the payload is then exposed to <10^−4^ *g* for a period up to 390 seconds. At this stage the rocket re-enters the atmosphere and following parachute-mediated deceleration the payload is recovered after landing on the Earth.

#### Construction of a versatile construct to visualise F-actin and α-tubulin

To construct pcDNA3.1-Lifeact-eGFP-IRES-mCherry-Tubulin, briefly, pLAGICT, a DNA sequence construct harboring a *Not*I restriction site, the Lifeact sequence, encoding a 17 amino acid long F-actin marker^63^ followed by a seven amino acid long linker (GDPPVAT) in frame with Lifeact, the cDNA sequence encoding eGFP, an internal ribosome entry site (IRES) sequence, the cDNA sequence encoding mCherry, a 7 amino acid long linker (SGLRSRA) in frame with mCherry, and the cDNA sequence encoding α-tubulin, followed by a *Xba*I restriction site was ordered from Genscript (Fig. 3A). The Lifeact-eGFP-IRES-mCherry-Tubulin sequence, provided in a pUC57 plasmid, was excised by digestion with *Not*I/*Xba*I (New England Biolabs), purified by gel extraction and ligated into the *Not*I/*Xba*I restriction sites of pcDNA3.1(+) (Invitrogen). Following transformation in ultracompetent *Escherichia coli* strain XL2-Blue (Stratagene) and overnight growth on LB/agar plates supplemented with ampicillin, colonies of interest were cultured overnight in 2 ml LB supplemented with ampicillin and plasmid DNA was purified from the remaining pellet by QIAprep spin column (Qiagen). Following digestion with *Not*I/*Xba*I plasmid DNA preparations harboring inserts of the expected size (~3.5 kb) were submitted to sequencing.

#### Generation of Lifeact-GFP expressing FTC-133 cells and cell culture procedure

To generate stable transfected cells FTC-133 cells were seeded in T75 flasks 75 cm^2^ (Sarstedt). The next day the cells were transfected with pLAGICT as previously described^64^. In brief, a total of 11.25 μg plasmid DNA and 33.75 μl X-tremeGENE 9 transfection (Roche) reagent in a total volume of 750 μl serum free medium was used according to the manufacturer’s instructions. Twenty-four hours post transfection, cells were seeded into P15 dishes (Greiner). Six to twelve hours later the medium was replaced with medium containing Neomycine (G418) 1.5 mg/ml (VWR). Approximately one week after the initiation of the selection procedure non-transfected cells were dead and several positive clones were harvested after an additional week of selection. Expression of Lifeact-GFP was validated by fluorescent microscopy.

For the PFC, the cells were cultivated either in T175 cell culture flasks (Sarstedt) until subconfluent monolayers were obtained or in *μ*-slide VI 0.4 ibiTreat slides (Ibidi) (Fig. 3D). One slide (each flight day) was taken to the aircraft for live-cell imaging with the FLUMIAS microscope, and another slide was investigated by fluorescence microscopy on ground (1 *g*) in the laboratory. Approximately one hour prior each of the three flights, a *μ*-slide (Fig. 3D) was mounted in the late access and fixation unit of the microscope (Supplementary Figure S1E) and brought to the aircraft together with the cell culture flasks. The late access and fixation unit containing the slide was loaded into the pre-heated FLUMIAS EM placed in a protective dome-shaped rack (Supplementary Figure S1C). The cell culture flasks were placed in the incubator, likewise pre-heated, on an experimental rack^1^.

Cells used for the TEXUS 52 mission were seeded into six *μ*-slides 28 hours prior to lift-off (Fig. 3D). Two hours before lift-off one *μ*-slide was selected and mounted in the late access and fixation unit and brought to the rocket, where it was loaded into the pre-heated FLUMIAS FM placed in a protective dome-shaped rack (Supplementary Figure S1D). The remaining *μ*-slides were used as ground control cells, hyper-*g* (1.8 *g*) control cells, and vibration (Vib) control cells, respectively (see below).

#### Hypergravity experiments

Experiments in hyper-*g* were conducted on the short-arm human centrifuge (DLR (German Aerospace Agency), Cologne, Germany) as described recently^1^. In brief, two portable incubators were placed in a swing-out device on the centrifuge to ensure that the cells were exposed to a correct vertical acceleration. The cells were exposed to a continuous hyper-*g* phase of 1.8 *g* for 2 h corresponding to the time period of 31 parabolas (31P). In parallel, the static 1 *g* controls were cultured in the laboratory. For real-time qPCR analysis n = 12 static 1 *g* controls and n = 12 1.8 *g* hyper-*g*-samples were collected. For imaging of cells exposed to hyper-*g* a *μ*-slide was placed in a Heraeus Omnifuge 2.0 RS centrifuge using a swing-out rotor and centrifuged at a continuous hyper-*g* phase of 1.8 *g* for 2 h. Imaging on the FLUMIAS FM was performed immediately after centrifugation.

#### Vibration experiments

T25 cell culture flasks containing 80–90% confluent monolayers were attached to a Vibraplex platform in an incubator at 37 °C with 5% CO_2_ and treated as previously described^5^. On the Vibraplex device the cells were exposed to vibrations comparable to those occurring during parabolic flights^65^. Frequencies ranging from 0.2 to 14 Hz were applied, corresponding to the three phases of a parabola, over 2 h, which corresponds to the total time period of 31 parabolas during a parabolic maneuver. For real-time qPCR analysis n = 5 static 1 *g* controls and n = 5 vibration samples were collected. For imaging of cells exposed to vibration the *μ*-slide was placed on a Vibraplex platform in an incubator at 37 °C and exposed to vibrations as described above. Imaging on the FLUMIAS FM was performed immediately after vibration.

#### RNA isolation and real-time qPCR analysis

qPCR was used to determine the expression levels of the genes of interest. The medium or the mixture of medium and RNA*later* was removed and replaced by 5–10 ml fresh RNAlater. Following storage at 4 °C the cells were scraped off by using cell scrapers (Sarstedt), transferred to 50 ml tubes, and pelleted by centrifugation (2500 *g* for 10 minutes at 4 °C). Total RNA was isolated according to the manufacturer’s instructions using the RNeasy Mini Kit (Qiagen). The quality and concentration of RNA were assessed spectrophotometrically by means of a NanoDrop instrument (Thermo Scientific). In all cases purified RNA had an A260/280 ration of >1.7.

cDNA obtained with the First-strand cDNA synthesis Kit (Fermentas) by applying 1 μg of total RNA in a 20 μl reverse transcription reaction was used for qPCR analysis in order to determine the expression levels of genes of interest. Primer Express software (Applied Biosystems) was applied to design appropriate primers with a Tm of ~60 °C (Supplementary Table S1). The assays were performed on a StepOnePlus Real-Time PCR System using the Power SYBR Green PCR Master Mix (both Applied Biosystems). The total reaction volume was 25 μl including 1 μl of template cDNA with a final primer concentration of 500 nM. PCR conditions were as follows: 10 minutes at 95 °C, 40 cycles of 30 seconds at 95 °C and 1 minute at 60 °C. The run was followed by a melting curve analysis step. If all amplicons showed a Tm similar to the one predicted by the Primer Express software, the PCR reactions were considered specific. Every sample was measured in triplicate, and relative quantification was obtained by means of the comparative C_T_ (∆∆C_T_) method. 18S rRNA was used as a housekeeping gene to normalize the expression data.

### Statistics

All statistical analyses were performed using SPSS 16.0 software (SPSS, Inc., Chicago, IL, USA). Data are presented as the mean ± SD. Statistical differences between two groups were evaluated using Mann-Whitney-U. P < 0.05 was considered statistically significant.

## Additional Information

**How to cite this article**: Corydon, T. J. *et al.* Alterations of the cytoskeleton in human cells in space proved by life-cell imaging. *Sci. Rep.* **6**, 20043; doi: 10.1038/srep20043 (2016).

## Supplementary Material

Supplementary Video S1

Supplementary Video S2

Supplementary Information
