# Supplementary material for: Alterations of the cytoskeleton in human cells in space proved by life-cell imaging
Source: Sci Rep. 2016 Jan 28;6:20043. doi: 10.1038/srep20043 (PMC4730242; doi:10.1038/srep20043)
Supplement: Supplementary Information [file srep20043-s3.pdf]

Alterations of the cytoskeleton in human cells in space proved by life-cell imaging

Thomas Juhl Corydon, Sascha Kopp, Markus Wehland, Markus Braun, Andreas Schütte,
Tobias Mayer, Thomas Hülsing, Hergen Oltmann, Burkhard Schmitz, Ruth Hemmersbach, Daniela Grimm

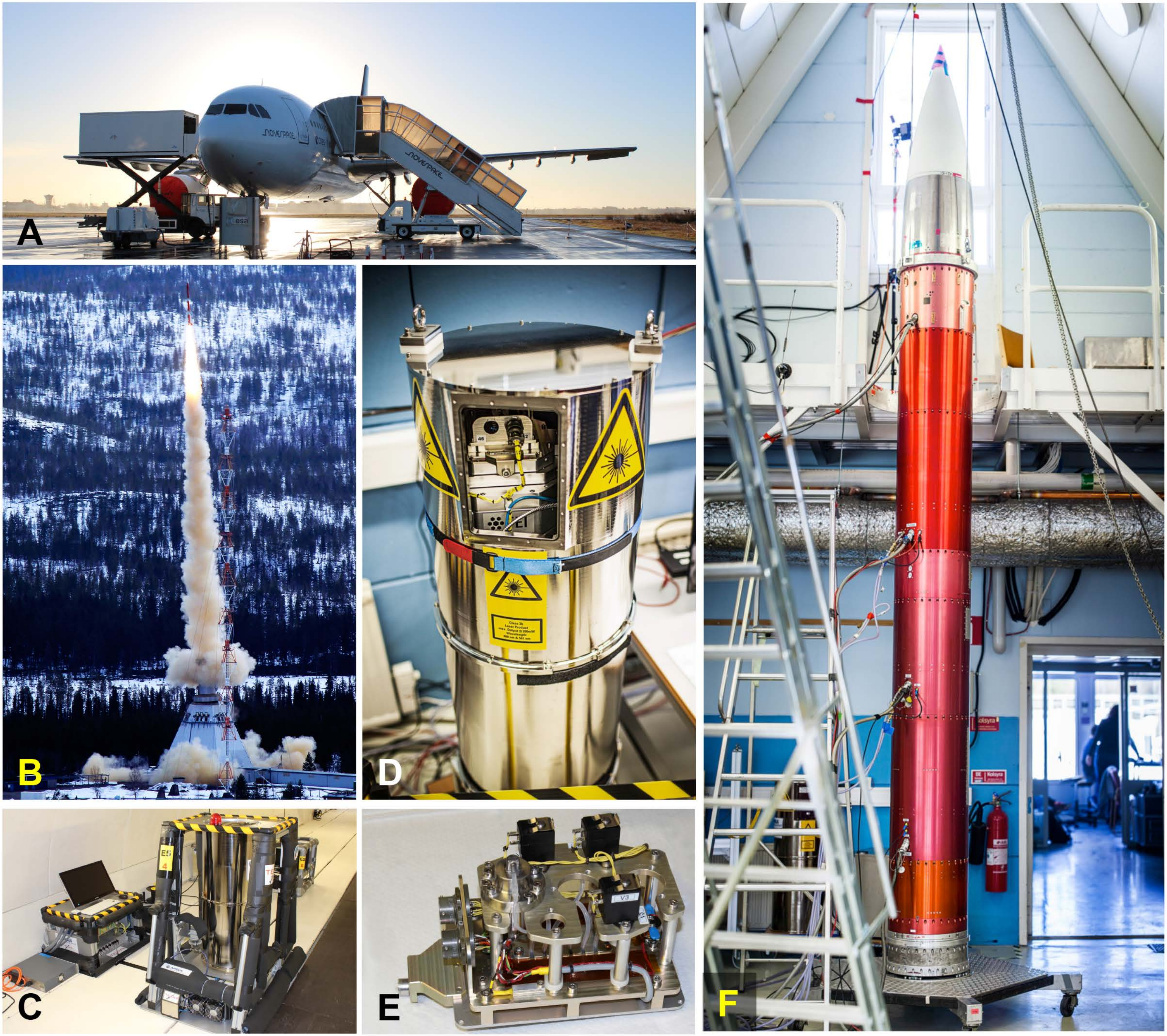

Alterations of the cytoskeleton in human cells in space proved by life-cell imaging

Thomas Juhl Corydon, Sascha Kopp, Markus Wehland, Markus Braun, Andreas Schütte, Tobias Mayer, Thomas Hülsing, Hergen Oltmann, Burkhard Schmitz, Ruth Hemmersbach, Daniela Grimm

Tables

Supplementary Table S1**: Primers used for quantitative real-time PCR**

Gene	***Primer Name***	***Sequence***
18S rRNA	18S-F	GGAGCCTGCGGCTTAATTT
	18S-R	CAACTAAGAACGGCCATGCA
ACTB	ACTB-F	TGCCGACAGGATGCAGAAG
	ACTB-R	GCCGATCCACACGGAGTACT
CPNE1	CPNE1-F	CAGAGCTGAGGGATGATGACTTC
	CPNE1-R	TTTCCAGGCTTCAGCATCAA
EZR	EZR-F	GAAGTGCACAAGTCTGGGTACCT
	EZR-R	CTCCCACTGGTCCCTGGTAAG
LCP1	LCP1-F	CTGACATCAAGGACTCAAAAGCTTATT
	LCP1-R	AATAACAACAGCAGGAACACCTTCT
LIMA1	LIMA1-F	TGGTAGCAGCGCAGAGGAA
	LIMA1-R	CCTAATGATGAGGTCCATTGC
MSN	MSN-F	GAAATTTGTCATCAAGCCCATTG
	MSN-R	CCATGCACAAGGCCAAGAT
RDX	RDX-F	GAAAATGCCGAAACCAATCAA
	RDX-R	GTATTGGGCTGAATGGCAAATT
SEPT11	SEPT11-F	TTGGAGACCAGATAAATAAAGATGACA
	SEPT11-R	CATGGTAGTTGAAGAGAGAACGTTTAA

All sequences are given in 5’-3’ direction.

Alterations of the cytoskeleton in human cells in space proved by life-cell imaging

Thomas Juhl Corydon, Sascha Kopp, Markus Wehland, Markus Braun, Andreas Schütte, Tobias Mayer, Thomas Hülsing, Hergen Oltmann, Burkhard Schmitz, Ruth Hemmersbach, Daniela Grimm

Online supplemental material legends

Supplementary Figure S1: The FLUMIAS-based platform for life-cell imaging under microgravity. (A) The parabolic flight plane. (B) Launch of the TEXUS 52 sounding rocket from Esrange, SSC, Kiruna. (C) The flight rack with the dome containing the FLUMIAS EM used for the PFC. (D) The protective dome containing the FLUMIAS FM used in the TEXUS 52 rocket. (E) The late access and fixation unit of the FLUMIAS microscope after integration of the μ-Slide. (F) The payload of TEXUS 52 containing the FLUMIAS FM.

Supplementary Video S1: Dynamics of the cytoskeleton changes during microgravity of the TEXUS 52 flight. Live-cell imaging video obtained during the approximately 125 second-long data collection period of the flight. Stress fiber formation and alterations of the actin filaments as well as cellular detachment are observed. White arrowheads indicate appearance of stress fibers. Purple arrowhead denotes cellular detachment.

Supplementary Video S2: Rocket flight video. An on-board camera situated under the payload captured the 15 minute-long parabolic flight of the TEXUS 52 rocket. Lift-off is at t = 00:07 seconds. At t = 01:07 minutes the rocket engine is released from the payload enabling the camera to film the flight including the microgravity phase, the re-entry (t = 07:40 minutes) into the atmosphere and the impact on Earth following a double-parachuted deceleration procedure (t = 14:32 minutes).
